# Diagnostic role of SPP1 and collagen IV in a rat model of type 2 diabetes mellitus with MASLD

**DOI:** 10.1038/s41598-024-64857-0

**Published:** 2024-06-17

**Authors:** Shan Xiao, Xiao Bei Wang, Ye Yang, Qin Wang

**Affiliations:** 1 Department of Endocrinology, People’s Hospital of Shenzhen Baoan District, The Second Affiliated Hospital of Shenzhen University, Shenzhen, 518100 Guangdong China; 2 Department of Neurology, Second Affiliated Hospital of Xinjiang Medical University, Urumqi, 830063 Xinjiang China; 3 Department of Geriatrics and Cadre Ward, Second Affiliated Hospital of Xinjiang Medical University, No. 38, South Lake East Road North Second Lane, Shuimogou District, Urumqi, 830063 Xinjiang China

**Keywords:** Biotechnology, Diseases, Medical research

## Abstract

Type 2 diabetes mellitus combined with metabolic dysfunction-associated steatotic liver disease (MASLD) leads to an increasing incidence of liver injury year by year, and patients are at a significantly higher risk of developing cirrhosis or even liver failure. No drugs have emerged to specifically treat this disease. The aim of this study is to investigate the mechanisms and causative hub genes of type 2 diabetes combined with MASLD. The data were obtained through the GEO platform for bioinformatics analysis and validated by in vitro experiments to find the causative targets of type 2 diabetes mellitus combined with MASLD, which will provide some theoretical basis for the development of future therapeutic drugs. GSE23343 and GSE49541 were downloaded from the Gene Expression Omnibus (GEO) database to identify differentially expressed genes (DEGs) in type 2 diabetes mellitus combined with MASLD for functional enrichment analysis. And STRING database and Cytoscape software were used to construct Protein–Protein Interaction (PPI) and hub gene networks. And GO (gene ontology, GO) analysis and KEGG (Kyoto encyclopedia of genes and genomes, KEGG) enrichment analysis were performed on target genes. A total of 185 co-expressed DEGs were obtained by differential analysis, and 20 key genes involved in the development and progression of type 2 diabetes were finally screened. These 20 key genes were involved in 529 GO enrichment results and 20 KEGG enrichment results, and were mainly associated with ECM–receptor interaction, Focal adhesion, Human papillomavirus infection, PI3K-Akt signaling pathway, and the Toll-like receptor signaling pathway. A total of two target genes (SPP1, collagen IV) were found to be highly correlated with type 2 diabetes mellitus combined with MASLD. Real time PCR results showed that there was a significant difference in SPP1 and collagen IV mRNA expression among the three groups (P < 0.05). SPP1 and Collagen IV may be candidate biomarkers for type 2 diabetes mellitus combined with MASLD, as verified by bioinformatics screening and in vitro experiments. Our findings provide new targets for the treatment of type 2 diabetes combined with MASLD.

## Introduction

Type 2 diabetes mellitus (T2DM) is a chronic metabolic disease characterised by insulin resistance and hyperglycaemia, and metabolic dysfunction-associated steatotic liver disease (MASLD) is common in patients with type 2 diabetes mellitus, with a prevalence of MASLD as high as 75% in patients with T2DM. MASLD is a metabolic disorder associated with sedentary lifestyle, insulin resistance^1^, and genetic and environmental factors^2^. Recent studies have shown that T2DM is an independent risk factor for MASLD^3^ and the combination of MASLD also increases the risk of macrovascular and microvascular complications of diabetes. Insulin resistance is not only a feature of type 2 diabetes, but also a key pathogenic driver of MASLD, and improving hepatic steatosis is effective in preventing the development of T2DM. Since type 2 diabetes is defined by high blood glucose levels, insulin resistance, and impaired islet cell function, type 2 diabetes can put patients with MASLD at increased risk for developing diabetes because both type 2 diabetes and MASLD are often associated with abnormalities in the expression of glucose metabolism and lipid metabolism^4^. Type 2 diabetes and its associated comorbidities, including visceral obesity, hypertension, and dyslipidaemia, may accelerate the transition from MASLD to metabolic dysfunction-associated steatohepatitis (MASH) and progression of liver disease to cirrhosis. On the other hand, MASLD may diminish hepatic insulin sensitivity in diabetic patients, which may further diminish the control of glucose metabolism^5^. Therefore, it is necessary to actively diagnose and treat type 2 diabetes mellitus with MASLD in order to prevent the progression of type 2 diabetes mellitus with MASLD to steatohepatitis (MASH), cirrhosis of the liver, and ultimately hepatocellular carcinoma^6^.

In the present study, we aimed to use a bioinformatics approach to explore the potential search for relevant biomarkers and pathogenesis of type 2 diabetes mellitus combined with MASLD, which could identify new diagnostic and therapeutic targets for patients with type 2 diabetes mellitus combined with MASLD. Using the GEO public database, we analysed DEGs in biopsies from patients with type 2 diabetes and subjects with normal glucose tolerance, and screened for disease key genes through protein interactions networks. Then, in order to analyse the main biological functions and signalling pathways of the key genes, GO and KEGG enrichment analyses were performed to further explore the signalling pathways associated with the disease, to explore the molecular mechanisms of the development of type 2 diabetes mellitus combined with MASLD and validate them using real-time fluorescence quantitative PCR to verify the results of the bioinformatic analyses even further.

Currently type 2 diabetes mellitus combined with MASLD therapeutic strategies are limited to symptomatic treatments, relying mainly on glucose-lowering and lipid-lowering drugs. However, the therapeutic drawbacks of these drugs are the lack of precision therapy and aetiological treatment, which leads to poor results in the treatment of diabetes mellitus combined with MASLD, in addition to the fact that not all patients can be effectively treated due to the hereditary nature and complexity of the disease, therefore, it is extremely urgent to search for new targets for the treatment of type 2 diabetes mellitus combined with MASLD. Currently, there are no approved drugs for the treatment of MASLD and MASH, so this study aims to provide some data to support the pathogenesis and pharmacological studies of diabetes mellitus combined with MASLD. In our current study, we found that: SPP1, collagen IV may be considered as potential candidate targets for the treatment of diabetes combined with MASLD.

## Materials and methods

### Data collection and pre-processing

The National Center for Biotechnology Information (NCBI) Gene Expression Omnibus database (https://www.ncbi.nlm.nih.gov/geo/) was used to obtain gene expression profiling data related to type 2 diabetes mellitus and MAFLD via the GEOquery package [version 2.54.1]^7^. Downloaded from the GEO database (https://www.ncbi.nlm.nih.gov/U) 2 separate GeneChip datasets GSE23343 and GSE49541. The dataset, GSE23343, includes liver tissue samples from 10 patients with type 2 diabetes and 7 subjects with normal glucose tolerance. Within the GSE49541 dataset, there were 72 liver tissue samples in the set, of which 40 were mild MASLD (fibrosis stage 0–1) and 32 were advanced MASLD (fibrosis stage 3–4). Data were standardised by limma [3.52.2]^8^. Principal component analysis (PCA) was performed on the standardised dataset using R (version 3.6.3). PCA plots were drawn using ggplot2 [3.3.6] to see clustering between sample groups.

### Identification of DEGs associated with type 2 diabetes combined with MASLD

The samples in GSE23343 and GSE49541 were extracted, and the samples in them were analysed for differences using limma [3.52.2]^8^ respectively, to obtain the differentially expressed genes (DEGs) between the livers of the diabetic patients and the livers of the subjects with normal glucose tolerance, and the results were de-emphasised. The FDR was used to correct the q-value for multiple hypothesis testing. log2FC| > 0.263, P < 0.05 was statistically significant. Subsequently, in order to better understand the DEGs, R (4.2.1) version “ggplot2 [3.3.6]” was applied to the obtained differentially expressed genes to plot volcano maps for DEmRNAs and “ComplexHeatmap [2.13.1]” to plot heatmaps for DEmRNAs, respectively plotted the heat map^9^. Finally, “VennDiagram [version 3.6.3]” and “ggplot2 [3.3.6]” were used to plot the Venn diagram of GSE23343 and GSE49541 co-intersecting genes.

### GO enrichment and KEGG signalling pathway enrichment analysis

The above DEGs were extracted and analysed for gene ontology (GO) function enrichment in the background of Homo sapiens using DAVID online database (https://david.ncifcrf.gov/). We provided the required GO function enrichment data, annotated and classified the genes according to their functions: biological process (BP), cellular component (CC), and molecular function (MF), and also Kyoto encyclopedia of genes and genomes (KEGG) signalling pathway enrichment analysis was used to discover the biological pathways that may be involved^10–12^. p < 0.05 and FDR < 0.2 were considered statistically significant to screen the major enriched functions and pathways of differential genes^13^. The clusterProfiler package [version 3.14.3] was used for enrichment analysis^14^. The GOplot package [version 1.0.2] was used to calculate z score values^15^ and finally to plot the bubble plots.

### Network analysis of protein–protein interaction (PPI) of common DEGs

The STRING database (https://string-db.org/) was used to present and evaluate the PPI network^16^. The common DEGs screened in this study were imported into STRING, and the potential connections between these DEGs could be further explored by the STRING analysis tool. The common differentially expressed genes were analysed for protein interactions networks using Cytoscape (version 3.9.1) with visualisation and association analysis^17^. Subsequently, the degree algorithm of CytoHubba plugin was applied to mark them as hub genes, and the top 20 genes at key positions in the PPI network were screened^17,18^. The potential hub genes were also analysed by GO and KEGG^13,14^.

## Research and methods

### Research design and methods

All procedures were approved by the Animal Ethic Committee of Animal Experimental Centre of Xinjiang Medical University (Xinjiang, China; IACUC-20181020-01). The experiments were completed in the Animal Experimental Center of Xinjiang Medical University and the laboratory of metabolic diseases of the clinical research institute of Xinjiang Medical University (Xinjiang, China). The experiments were completed in the Animal Experimental Center of Xinjiang Medical University and the laboratory of metabolic diseases of the clinical research institute of Xinjiang Medical University (Xinjiang, China).

### Preparation of T2DM combined with MASLD rat model

Thirty healthy clean-grade male Sprague–Dawley rats, aged 6–8 weeks, weighing 180–220 g, were provided by Wuhan Yunkron Technology Company. The animal production and use license number is DCXR(E)2018-0021. SYXK (E) 2013-0069, respectively. After 1 week of normal feeding, high-fat and high-sugar diets and regular diets were prepared according to the formula. Twenty rats were fed with high-fat and high-sugar diet, high-fat and high-sugar diet feed formula: 10% lard, 20% sucrose, 2% cholesterol, 60% ordinary feed, 8% egg yolk powder(Jiangsu Xietong Pharmaceutical Bio-engineering Co., Ltd.) and 10 rats in normal control group were fed with normal diet. Twenty rats were fed a 100 g high-fat and high-sugar diet daily with free access to food and water. Corn oil 5 mL/kg was given by gavage on an empty stomach at 8:00 am every morning. At the end of the 12th week, 10 rats in the model group were given intraperitoneal injection of streptozotocin (STZ) (30 mg/kg) overnight fasting, and the other 10 rats in the model group were not given STZ injection. Ten control rats fed a regular diet were injected with the corresponding volume of citrate buffer. Blood samples were collected from the tail vein of 10 rats fed a high-fat and high-sugar diet and injected with STZ 3 days later, and random blood glucose was ≥ 16.7 mmol/L. Blood glucose was monitored after 2 weeks, and fasting blood glucose was detected on the 2nd, 4th, 6th, 8th, 10th, 12th and 14th days. At the 14th week, 4 rats in each group were randomly selected to complete liver ultrasound, and fatty liver results were formed to determine the success of modeling. Finally, the rats were divided into 3 groups: (1) T2DM + MASLD group (n = 10); (2) High-fat and high-glucose group (n = 10); (3) normal control group (n = 10); After 15 weeks, the rats were sacrificed under anesthesia, and the livers were collected, rinsed with normal saline at 4 °C, weighed and placed in preservation solution and stored in a refrigerator at − 80 °C.

### Quantitative real-time PCR

Real-time fluorescence quantitative PCR (Polymerase Chain Reaction): 0.15 g of rat liver tissue was taken from each group, and total RNA was extracted by the Trizol method. cDNA was reverse-transcribed into the corresponding cDNA in accordance with the reverse-transcription kit, and then subjected to real time Polymerase Chain Reaction (PCR), and primer sequences are shown in Table 1, Chain Reaction), primer sequences are shown in Table 3. SPP1, collagen IV mRNA expression level: PCR reaction total system 20 μL, PCR amplification conditions: denaturation at 95 °C for 10 min, annealing at 60 °C for 1 min, extension at 95 °C for 15 s, 40 cycles, each sample set up 3 replicate wells, internal reference GAPDH. The results of the experiments were analysed by Bio-Rad Fluorescence Quantitative Analysis. The results of the experiments were read by Bio-Rad fluorescence quantitative analysis software, and the quantitative calculation of the mRNA expression levels of SPP1 and collagen IV in each group was expressed by 2^−∆∆Ct^ (CT is the number of cycles). The main observation indexes were SPP1, collagen IV mRNA expression levels in rat liver tissues in each group.

**Table 1:** Sequences of real-time fluorescence quantitative PCR primers.

Rat GAPDH F	ACTCTACCCACGGCAAGTTC
Rat GAPDH R	tggggtttcccgttgatgacc
Rat SPP1 F	CCAGCCAAGGACCAACTACA
Rat SPP1 R	AGTGTTTGCTGTAATGCGCC
Rat collagen IV F	caccctgaactcaagagcgg
Rat collagen IV R	tgcatgtttctccggtttcc

### Study approvals

All animals received humane care according to criteria outlined in the Guide for the Care and Use of Laboratory Animals, prepared by the National Academy of Sciences, and published by the National Institutes of Health. All animals received humane care according to criteria outlined in the Guide for the Care and Use of Laboratory Animals, prepared by the National Academy of Sciences, and published by the National Institutes of Health. Housing and husbandry conditions were approved by the Institutional Animal Care and Use Committee before initiating the studies. All in vivo experiments were carried out according to Animal Research: Reporting of In Vivo Experiments guidelines. All in vivo experiments were carried out according to Animal Research: Reporting of In Vivo Experiments guidelines.

### Statistics

The experimental data were processed using SPASS 25.0 statistical software, and the measurement data were expressed as x ± s. One-way ANOVA was used for comparison among three groups, and Tukey was used for comparison between two groups, with *P* < 0.05 or *P* < 0.01 as the significance of the difference.

## Results

### Hub gene screening for type 2 diabetes combined with MASLD

**Figure 1:**
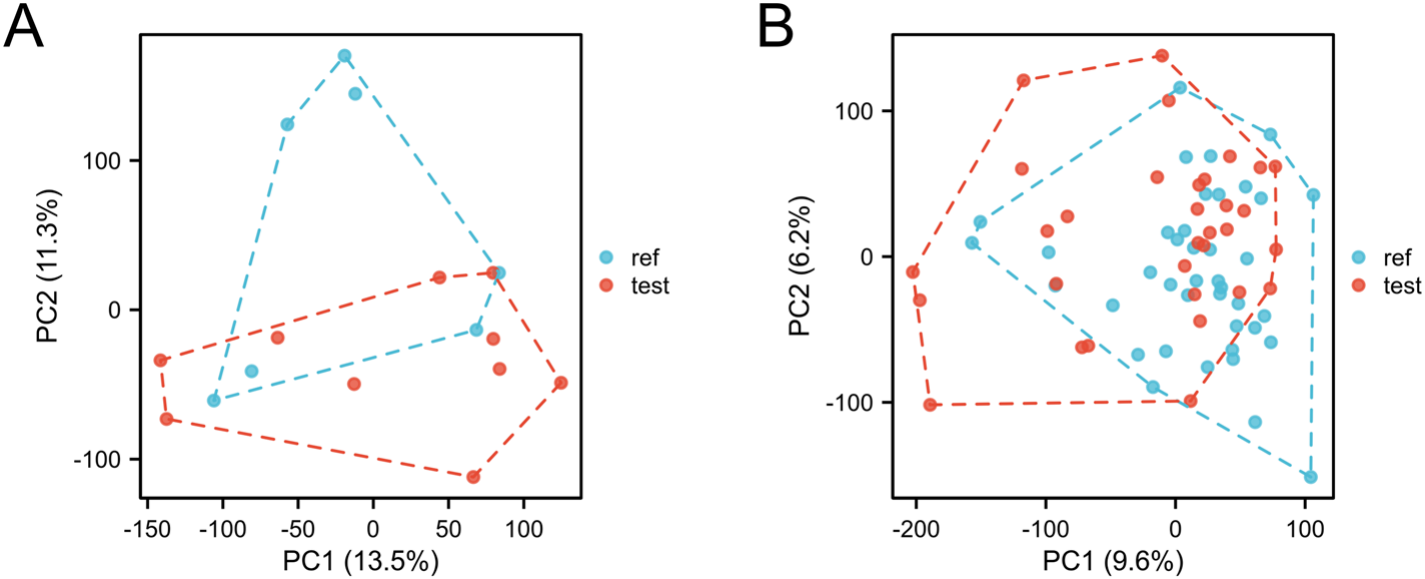
Principle component analysis (PCA) of GSE23343 and GSE49541. (**A**) The two main components contributed 13.5% and 11.3%. PCA of GSE23343. (**B**) The two main components contributed 6.2% and 9.6%. PCA of GSE49541.

**Figure 2:**
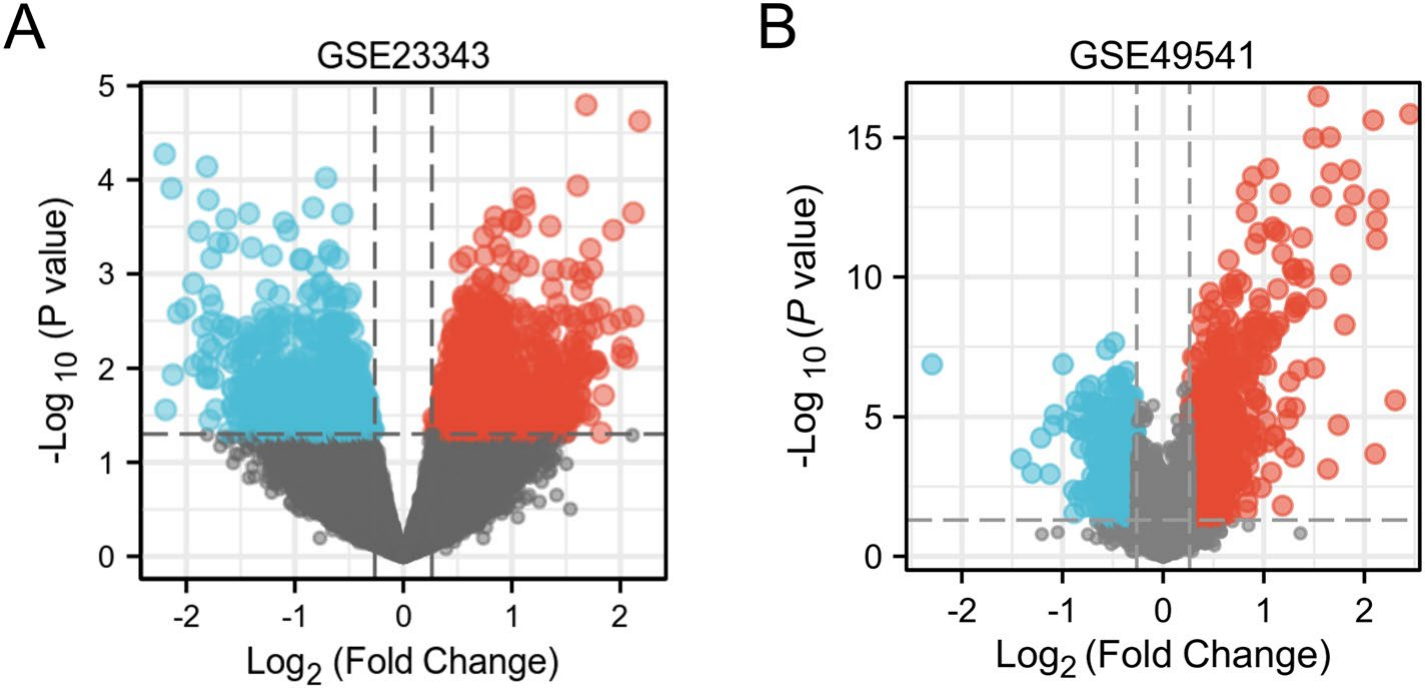
Volcano map of GSE23343 (**A**) and GSE49541 (**B**). |log2FC| > 0.263, P < 0.05 (red was the upregulated gene, blue was the downregulated gene and grey was the undifferentiated gene).

**Figure 3:**
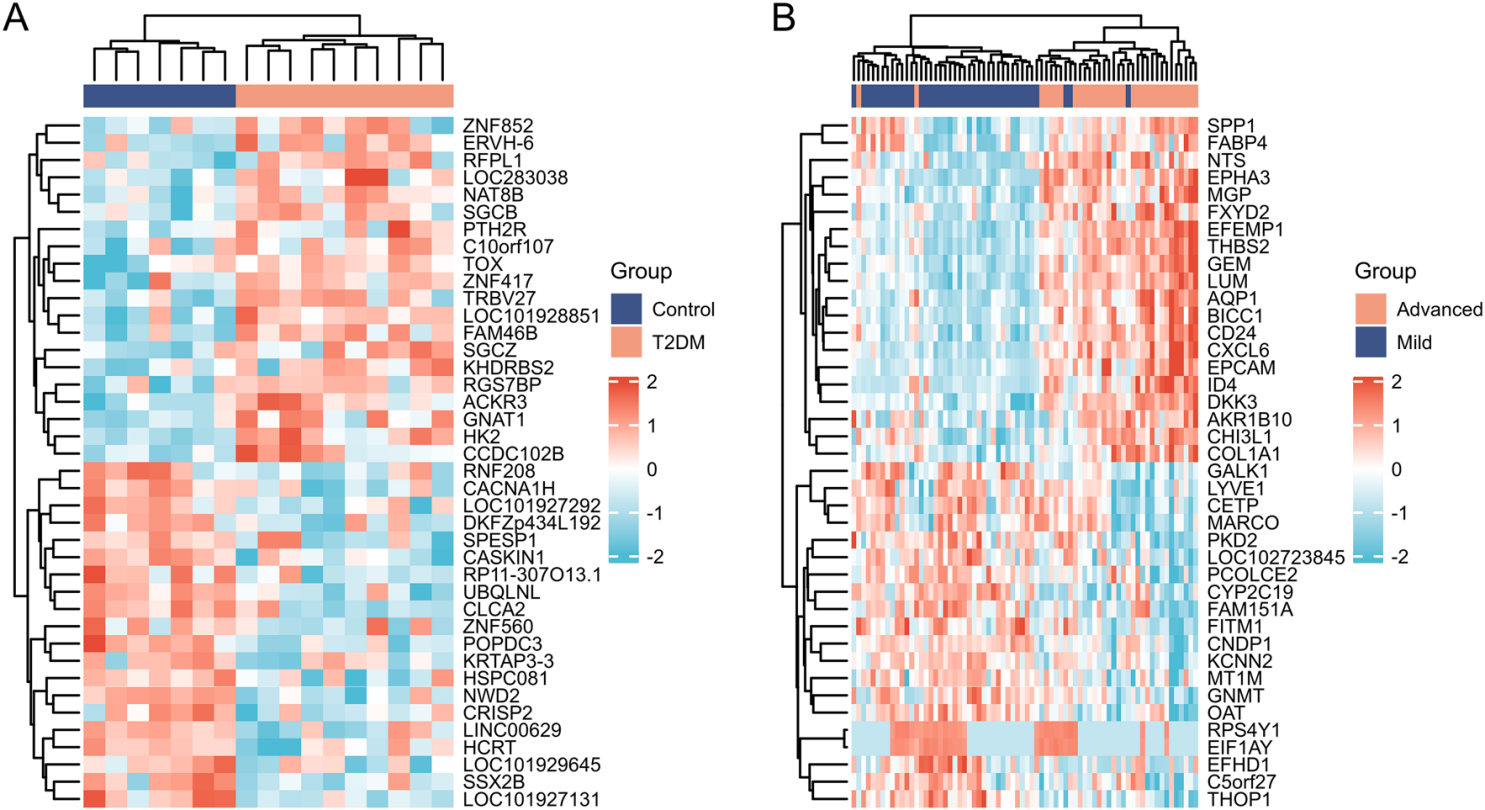
Hierarchical clustering heatmap of GSE23343 (**A**) and GSE49541 (**B**) (Note: red was upregulated. and blue was down regulated. Blue and orange sample bars below indicate the Mild and Advanced samples).

**Figure 4:**
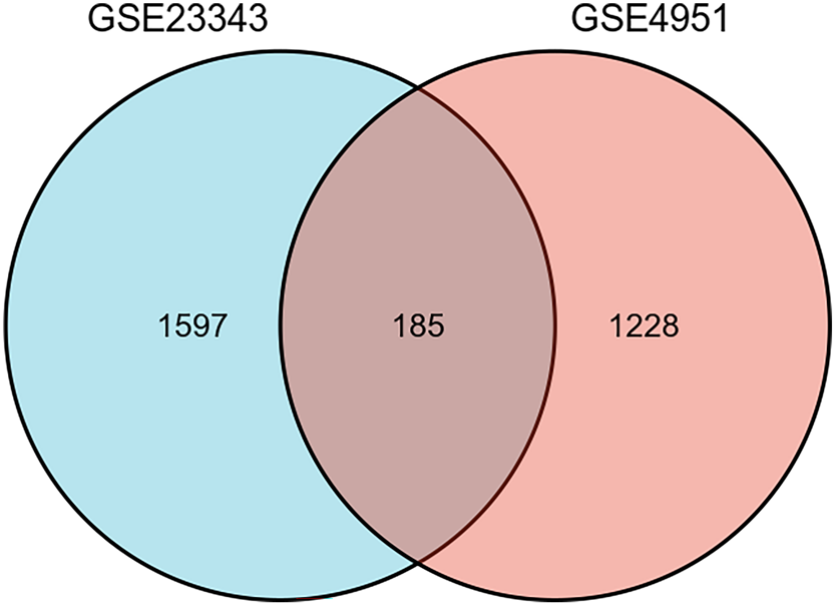
Venn diagram: the intersection of GSE23343 and GSE49541.

For screening DEGs, dataset GSE23343 was downloaded from GEO, which included a total of 10 liver samples from patients with type 2 diabetes mellitus and 7 liver counterpart samples from subjects with normal glucose tolerance, and dataset GSE49541, in which a total of 72 liver tissue samples were included, of which 40 were mild MASLD (fibrosis stage 0–1, hereafter referred to as Mild) and 32 cases of advanced MASLD (fibrosis stage 3–4, hereafter referred to as Advanced). The 2 datasets were normalised separately. PCA principal component analysis was performed on GSE23343 and GSE49541, and scatter plots were used to demonstrate clustering, with significant differences between subgroups (Fig. 1A,B). The volcano plot showed that dataset GSE23343 identified 21,654 differentially expressed genes, of which 11,180 genes were up-regulated and 10,473 genes were down-regulated (Fig. 2A). The dataset GSE49541 identified 21,655 differentially expressed genes, of which 9497 genes were up-regulated and 12,155 genes were down-regulated (Fig. 2B). Among them, the top20 genes each up- and down-regulated in GSE23343 and GSE49541 are shown on the heatmap (Fig. 3A,B). Finally, according to the screening condition |log2FC| > 0.263, P < 0.05, 1782 expression differential genes were screened from the GSE23343 dataset and 1413 expression differential genes from the GSE49541 dataset, and the creation of Wayne plots to take the overlapping genes showed that 185 overlapping DEGs were generally expressed differently (Fig. 4).

### Results of GO and KEGG enrichment analysis

**Figure 5:**
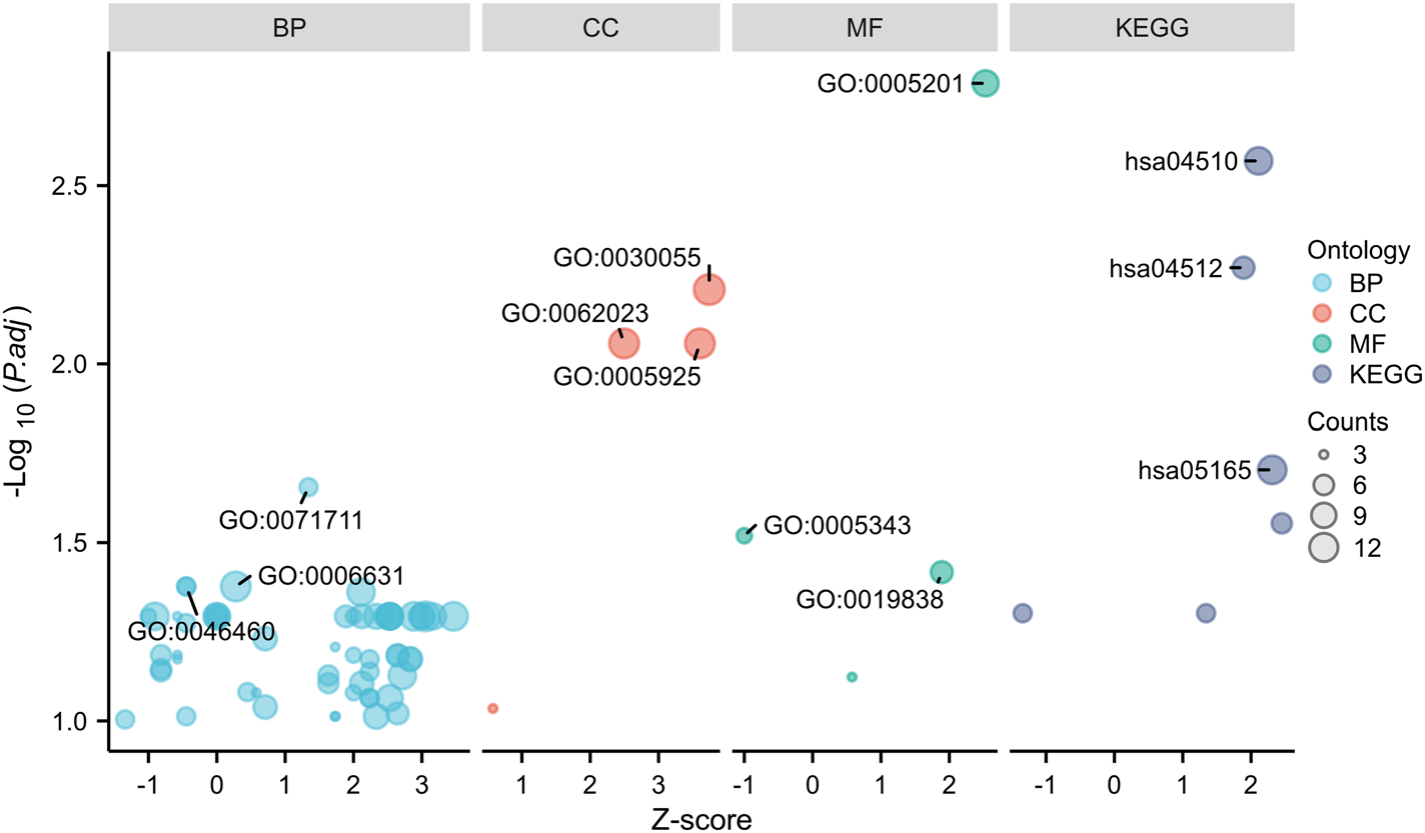
Functional enrichment analysis of 185 co-expressed differential genes. GO and KEGG enrichment analyses, GO analyses include BP (biological process), CC (cellular component), MF (molecular function). Z-scores are defined as (up-regulated genes − down-regulated genes)/total genes.

The 185 DEGs were subjected to DAVID-based GO biological process and KEGG signalling pathway enrichment annotation analysis, which satisfied the conditions of p. adj < 0.05 and q value < 0.2 significant enrichment, with a total of 58 for BP, 4 for CC, 4 for MF and 6 for KEGG. After arranging them according to FDR values from smallest to largest, they were visualised as in (Fig. 5). These DEGs were significantly enriched in biological processes such as fatty acid metabolic process, neutral lipid biosynthetic process, acylglycerol biosynthetic process, regulation of cell morphogenesis, I-kappaB kinase/NF-kappaB signalling, fatty acid oxidation, response to tumour necrosis factor, cytokine-mediated signaling pathway, triglyceride biosynthetic process, etc. In KEGG enrichment analysis, DEGs were involved in Focal adhesion, ECM–receptor interaction, Human papillomavirus infection, Small cell lung cancer, p53 signaling pathway, PPAR signaling pathway. signaling pathway.

### Construction of PPI network and identification of hub genes

**Figure 6:**
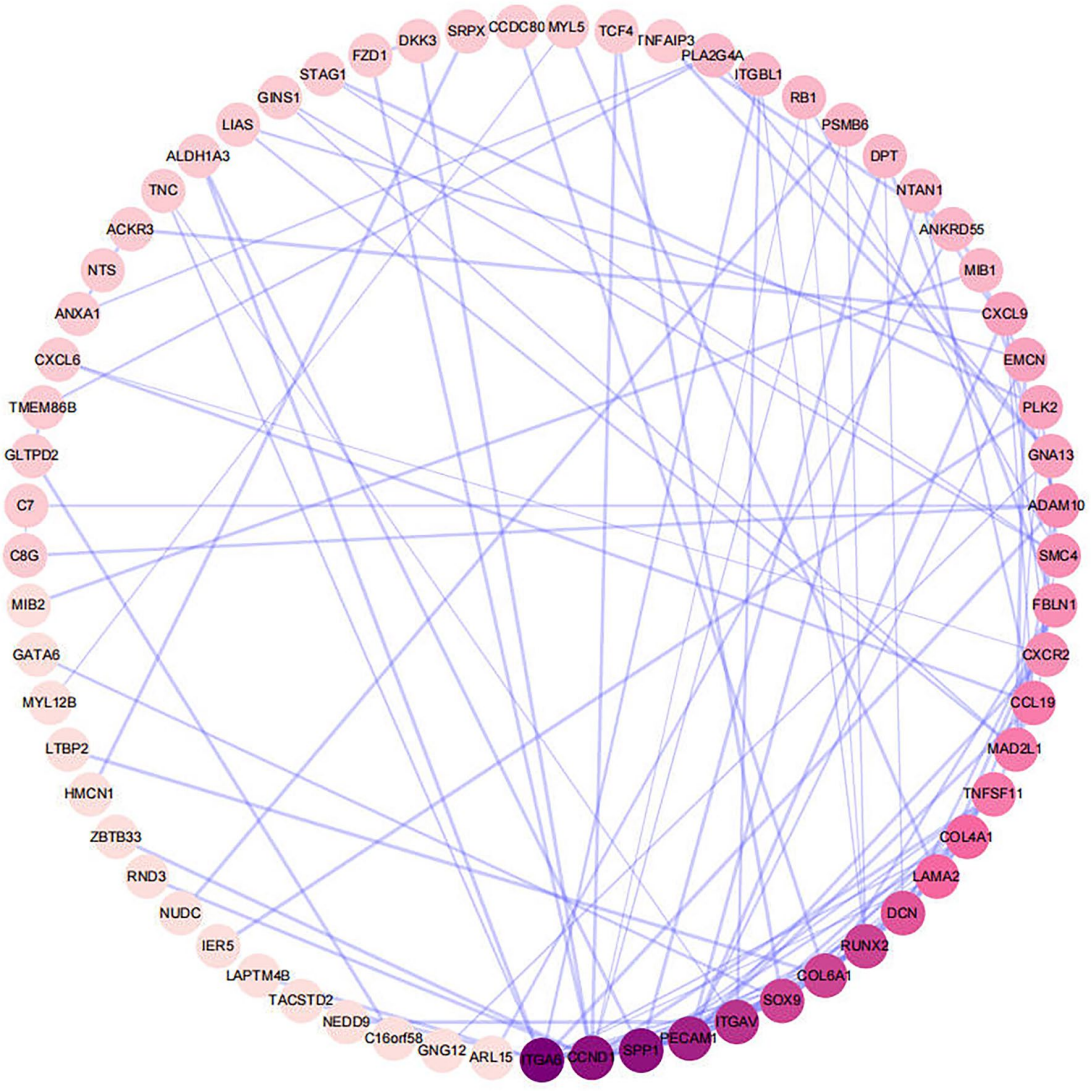
PPI network of up-regulated co-expressed genes constructed based on Cytoscape. Nodes represent proteins and edges represent protein interactions. The colour depth of the nodes is the DEGREE value and the colour depth of the edges is the COMBINED-SCORE value, both indicating their importance in the network.

**Figure 7:**
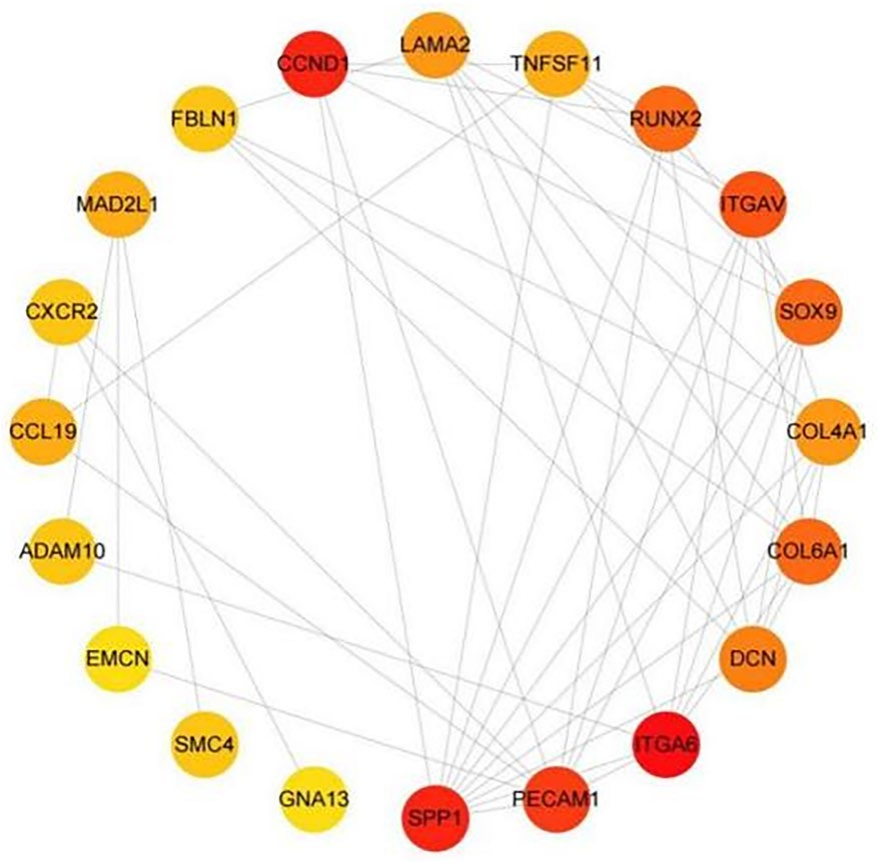
Gene identification of the first 20 Hubs.

**Figure 8:**
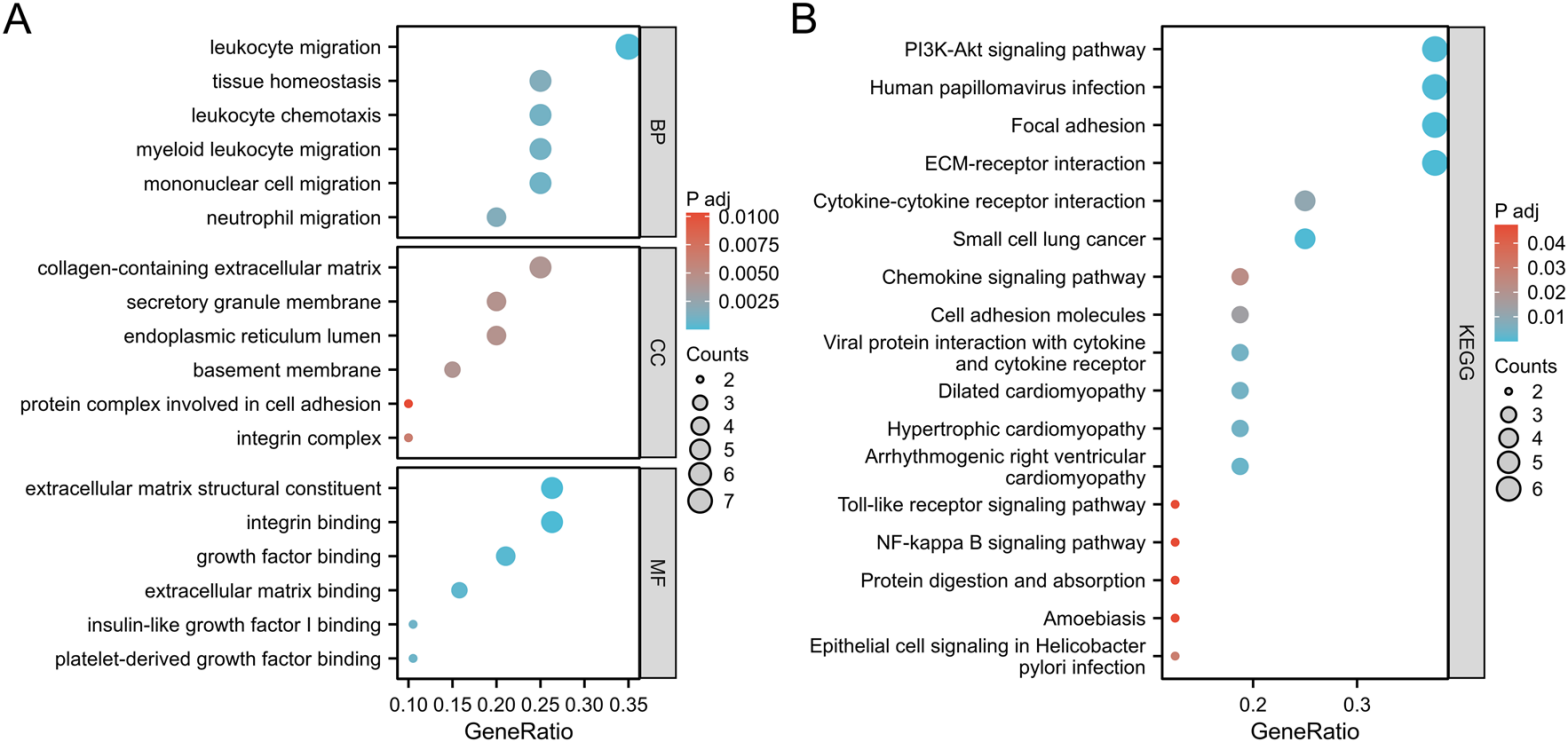
Results of GO and KEGG functional enrichment analysis of TOP20 Hub genes. (**A**) GO analysis included BP (biological process), CC (cellular component), and MF (molecular function). (**B**) Results of KEGG functional enrichment analysis.

**Figure 9:**
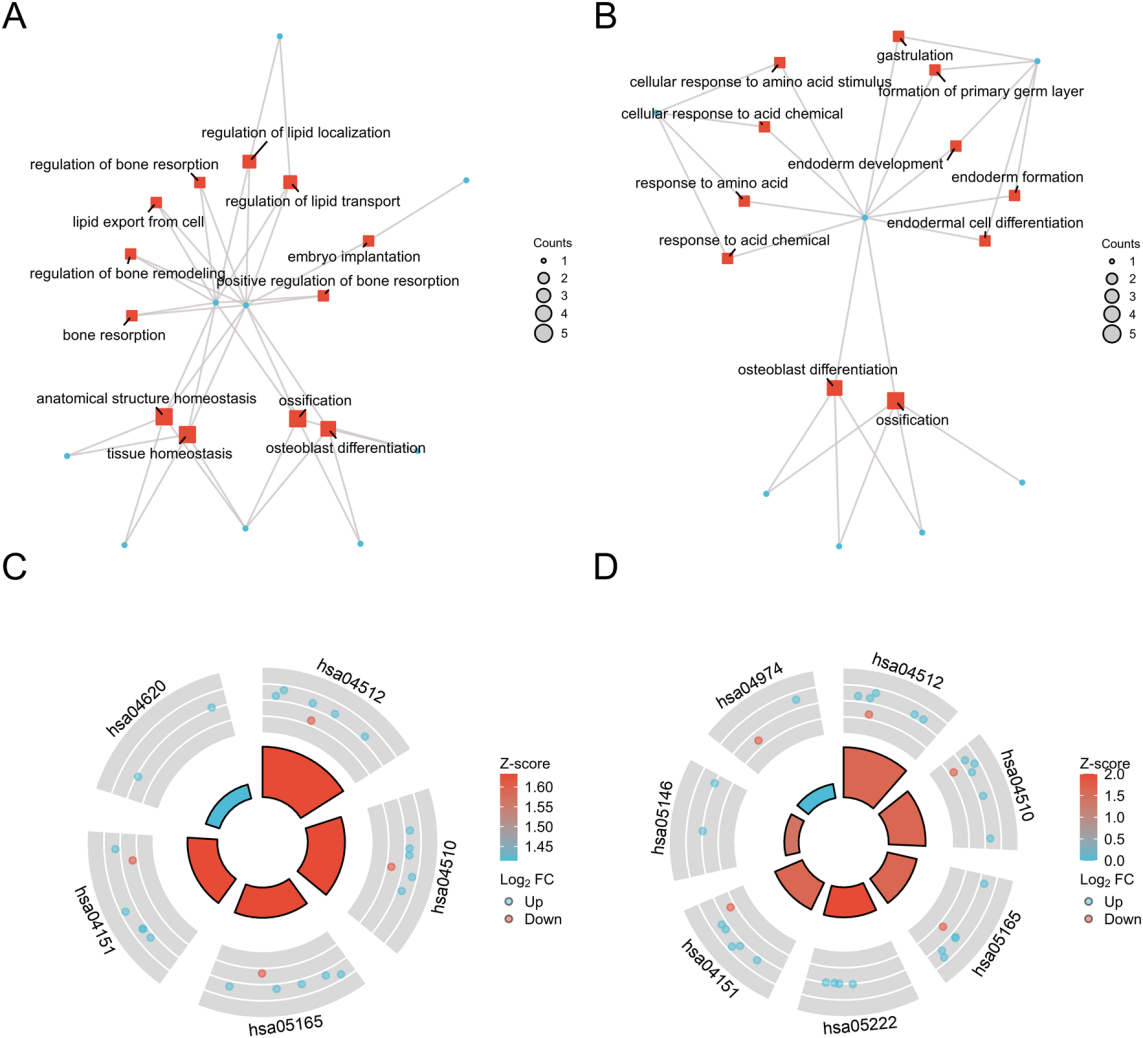
Functional enrichment analysis results of the 2 hub genes. (**A**) GO analysis results of SPP1. (**B**) GO analysis results of collagen IV. (**C**) KEGG enrichment analysis results of SPP1. (**D**) KEGG enrichment analysis results of collagen IV.

To understand the interactions between differential genes, a PPI network was constructed for co-expressed DEGs using STRING (Fig. 6). The results were imported into Cytoscape v.3.8.2 software, and the genes in this network were sorted according to their degree values using the cyto-Hubba plugin to identify the top 20 hub genes with the highest degree among them. These hub genes were ITGA6, SPP1, PECAM1, ITGAV, COL6A1, RUNX2, DCN, SOX9, Collagen IV, LAMA2, MAD2L1, CCL19, ADAM10, FBLN1, CXCR2, TNFSF11, EMCN, SMC4, CXCL9, NTAN1 as in Fig. 7. Subsequently, GO and KEGG enrichment analyses of these 20 pivotal genes showed that most of the 20 key genes were enriched in biological processes and pathways associated with the onset and progression of type 2 diabetes combined with MASLD as in (Fig. 8A,B). Among them, SPP1 was associated with tissue homeostasis, anatomical structure homeostasis, regulation of lipid transport and localisation, lipid export from cell (Fig. 9A), and also involved in ECM (Fig. 9B), and also involved in ECM–receptor interaction, Focal adhesion, Human papillomavirus infection, PI3K-Akt signaling pathway, Toll-like receptor signaling PI3K-Akt signaling pathway, Toll-like receptor signaling pathway (Fig. 9C). Collagen IV is mainly involved in biological processes such as extracellular matrix, structure and structure organisation, cellular response to amino acid stimulus, cellular response to acid chemical (Fig. 9C). to acid chemical and other biological processes (Fig. 9B, Table 2), meanwhile, collagen IV was mainly enriched in ECM–receptor interaction, Focal adhesion, Human papillomavirus infection, Small cell lung cancer, PI3K-Akt signaling pathway, Amoebiasis, and Protein digestion and absorption (Fig. 9D, Table 3).

**Table 2:** Results of KEGG enrichment analysis of SPP1.

Ontology	ID	Description	GeneRatio	pvalue	p. adjust	z score
KEGG	hsa04512	ECM–receptor interaction	6/16	9.7e−09	4.65e−07	1.632993
KEGG	hsa04510	Focal adhesion	6/16	1.35e−06	3.23e−05	1.632993
KEGG	hsa05165	Human papillomavirus infection	6/16	2.41e−05	0.0003	1.632993
KEGG	hsa04151	PI3K-Akt signaling pathway	6/16	3.53e−05	0.0003	1.632993
KEGG	hsa04620	Toll-like receptor signaling pathway	2/16	104/8164	0.0172	0.0475

**Table 3:** Results of KEGG enrichment analysis of collagen IV.

Ontology	ID	Description	GeneRatio	BgRatio	p value	p. adjust
KEGG	hsa04512	ECM–receptor interaction	6/16	88/8164	9.7e−09	4.65e−07
KEGG	hsa04510	Focal adhesion	6/16	201/8164	1.35e−06	3.23e−05
KEGG	hsa05165	Human papillomavirus infection	6/16	331/8164	2.41e−05	0.0003
KEGG	hsa05222	Small cell lung cancer	4/16	92/8164	2.48e−05	0.0003
KEGG	hsa04151	PI3K-Akt signaling pathway	6/16	354/8164	3.53e−05	0.0003
KEGG	hsa05146	Amoebiasis	2/16	102/8164	0.0165	0.0475
KEGG	hsa04974	Protein digestion and absorption	2/16	103/8164	0.0169	0.0475

### Real-time fluorescence quantitative PCR experiments

**Figure 10:**
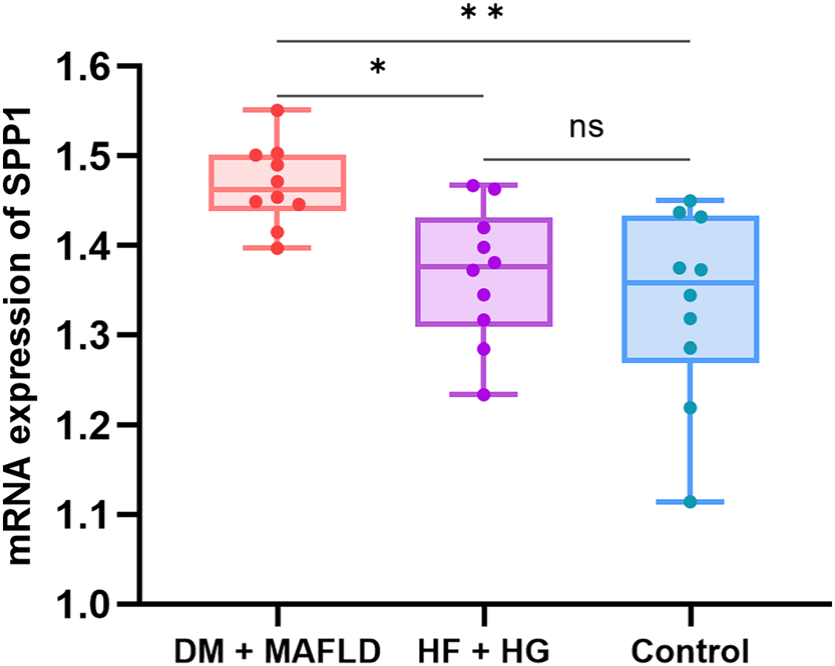
mRNA expression of SPP1.

**Figure 11:**
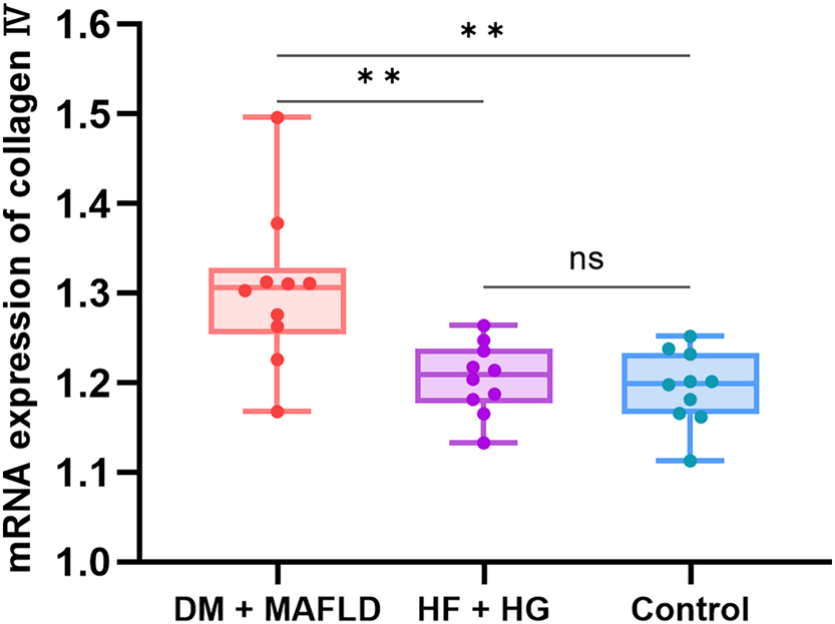
mRNA expression of collagen IV.

The real time PCR results showed that: One-way analysis of variance showed that there was a significant difference in SPP1 mRNA expression among the three groups (P < 0.05). Further comparison between the two groups using Tukey method suggested that the DM + MASLD group had a significant difference compared with the Control group (P < 0.01). There was significant difference between DM + MASLD group and HF + HG group (P < 0.05). The mean distribution between the HF + HG group and the Control group was not statistically significant (Fig. 10, Supplementary Table 1). One-way analysis of variance showed that there was a significant difference in the mRNA expression level of Collagen IV among the three groups (P < 0.05). Further comparison between the two groups using Tukey method suggested that the DM + MASLD group had a significant difference compared with the Control group (P < 0.01). There was significant difference between DM + MASLD group and HF + HG group (P < 0.01). The mean distribution between the HF + HG group and the Control group was not statistically significant. (Fig. 11, Supplementary Table 2).

## Discussion

As the prevalence of diabetes mellitus gradually climbs, the number of patients with diabetes mellitus combined with MASLD has also increased significantly. The 2018 Guidelines for the Prevention and Control of MASLD indicate that the prevalence of fatty liver in diabetic patients in China is 28–70%^19^. Globally, the prevalence of T2DM combined with MASLD is increasing year by year, and a recent meta-analysis shows that the global prevalence of this disease is about 55.48%, with regional differences, and the prevalence in East Asia is about 52.04%^20^. Patients with T2DM combined with MASLD are prone to impaired liver function and even cirrhosis, and at the same time, increase the risk of renal disease and cardiovascular disease such as coronary heart disease. It also increases the risk of kidney disease and cardiovascular disease such as coronary heart disease, which requires urgent attention. Since T2DM and MASLD share a common pathophysiological mechanism, namely insulin resistance, both diseases affect the development of the other. T2DM can exacerbate MASLD by promoting the progression of non-alcoholic hepatic steatosis or fibrosis, while MASLD can lead to macrovascular and microvascular complications in patients with T2DM^21^. The overall mortality rate for diabetic patients with MASLD was found to be as high as 58.5/103, which is significantly higher than the mortality rate for viral hepatitis B and C combined^20^. In addition, MASLD is an important cause of cryptogenic cirrhosis, with higher rates of liver-related complications and mortality^22^. Therefore early and effective treatment of T2DM in combination with MASLD will help to improve the quality of patient's survival and at the same time reduce the burden on health insurance. Although there has been an increasing amount of research on the diagnosis and treatment of diabetes in recent years, the understanding of the pathogenesis of type 2 diabetes mellitus combined MASLD is still very limited, and there is a lack of targeted pharmacological treatments. Therefore, it is particularly important to find the gold standard prevalence factors for type 2 diabetes mellitus combined with MASLD.

In the current study, we constructed a PPI network by analysing the GEO database datasets GSE23343 and GSE49541 for type 2 diabetes and MASLD, and screening 185 co-expressed DEGs. Twenty hub genes were also screened, and combined with GO and KEGG enrichment you analysis, the potential biomarkers and biological pathways in T2DM combined with MASLD were finally identified. We obtained 2 target genes, namely SPP1 and collagen IV, which may be the key genes of pathogenicity. This time, we validated the results by real-time fluorescence quantitative PCR experiments in diabetes combined with MASLD rat model, and obtained the following results: the mRNA content of SPP1 and collagen IV in diabetes combined with MASLD rat group were higher than that in the control group, which was consistent with the results of our bio-bioinformatics analysis, and once again verified the results of our bioconfidence analyses. Meanwhile, we also found that in the KEGG enrichment analysis, the two target genes, SPP1 and collagen IV, were mainly involved in the ECM–receptor interaction, Focal adhesion, Human papillomavirus infection, PI3K-Akt signaling pathway, Toll-Akt signaling pathway, and Toll-mediated protein expression. signaling pathway, Toll-like receptor signaling pathway.

Secreted phospho protein 1 (SPP1) is widespread in human tissues and organs and its expression is significantly upregulated in a variety of tumours^23–25^. Macrophage-positive SPP1-expressing colon cancer patients have shorter progression-free survival^26^. SPP1 is an important extracellular matrix component secreted by a variety of cells, including tumour cells, immune cells, fibroblasts, osteoblasts, smooth muscle cells, lymphocytes and epithelial cells. Up-regulation of SPP1 expression in tumour tissue and serum of a variety of tumours correlates with poor patient prognosis^27–29^. A study^30^ has shown that in lung adenocarcinoma, SPP1 regulates macrophage polarisation towards the M2 type, but the exact mechanism is unclear and has been less well studied in HCC. This study showed that the expression of SPP1 was higher in tumour tissues of patients with HCC hepatocellular carcinoma than in paracancerous tissues, and was elevated in patients with advanced BCLC (Barcelona Clinic Liver Cancer) stage, large tumour diameters, and multiple tumour foci in the liver, and was an independent prognostic factor in patients with hepatocellular carcinoma. The survival of patients with high expression of SPP1 was significantly lower than that of patients with hepatocellular carcinoma. The survival of patients with high SPP1 expression was shorter than that of patients with low SPP1 expression. Meanwhile, the expression of SPP1 was positively correlated with the number of M2 macrophages, suggesting that treatment targeting SPP1 may be a potential therapeutic option to improve the prognosis of HCC patients.

Expression of spp1 is associated with fibrosis and progression to MASH. In humans and mice, upregulation of bone marrow cell-derived spp1 is associated with progression to MASH. However, it is unclear whether the increase in spp1 in these cells is protective or deleterious. Studies have shown that spp1 accelerates the progression of MASLD^31^.

SPP1 has been less studied in diabetes combined with MASLD. In this study we found that SPP1 is involved in diabetes combined with MASLD. In this real-time fluorescence quantitative PCR experiment, there was also a clear and statistically significant high expression of SPP1 in the diabetes combined with MASLD group compared to the control group.

Collagen is a large family of glycoprotein molecules that are the main protein component of connective tissues, accounting for about 25% of the total protein in the body. All collagens are distributed in the extracellular matrix in a supramolecular structure of triple helical polypeptide chains. General proteins are double helical structures, and as a structural protein in the extra cellular matrix (ECM), collagen consists of three polypeptide chains that form a triple-stranded helical structure, or collagen domains, each of the three polypeptide chains is rotated left-handed to form a left-handed helical structure, which is then occluded by hydrogen bonds to form a strong right-handed superhelical structure. Reticular collagen includes collagen type IV, collagen type VIII, and collagen type X. Collagen type IV is the typical reticular collagen found in basement membranes and plays an important role in molecular filtration. collagen type VIII is localised in Descemet's membrane and in the subendothelial matrix of blood vessels, and collagen type X is localised in the proliferative zone of growth plate cartilage. The more classical collagens in pancreatic cancer are types COLI, COLIII, and COLIV. Collagen type IV is a member of the collagen family and is a major component of cellular basement membranes (BMs). And the main biological behaviour of malignant tumours is that cancer cells can break through BMs, invade and metastasize to adjacent or distant sites. As a major component of BMs, relevant studies have shown that type IV collagen plays an important role in the invasion and metastasis mechanism of multi-system malignant tumours and clinical diagnosis and treatment^32^. Fatty liver is a progressive disease, with 10–25% of patients developing cirrhosis or even hepatogenic death after 10 years.

Most researchers believe that excessive deposition of large amounts of ECM in the liver is the basis for the formation of hepatic fibrosis. Liver fibrosis is a dynamic process in which collagen is the most important component of ECM, and collagen deposition and degradation lead to disease progression and regression, respectively. Currently, studies have shown that high glucose significantly induces mesangial cell proliferation and extracellular matrix proteins, including fibronectin and collagen IV expression^33^. In addition, activation of Nrf2 suppressed high glucose-induced oxidative stress and expression levels of TGF-β1, fibronectin and collagen type IV in mesangial cells^34^.

High glucose and TGF-β1 synergistically induce collagen IV and VEGF production in podocytes. The high glucose-induced increase in collagen IV and VEGF proteins is mediated by the TGF-β system. By increasing TβRII expression, high glucose increases the response of podocytes to environmental levels of TGF-β^35^.

Collagen IV has been less studied in diabetes mellitus combined with MASLD. This time in our study collagen IV was found to be involved in diabetes combined with MASLD. In this real-time fluorescence quantitative PCR experiment, there was also a clear and statistically significant high expression in the diabetic combined MASLD group compared to the control group, which can be verified by western blot in later experiments.

There are some limitations of our study. First, this study was based on bioinformatics analyses of transcriptome profiles from public databases, which may differ from reality. Second, although the 2 genes screened have previously been reported to mediate type 2 diabetes and metabolism-related diseases, there is no direct evidence that they regulate the onset, progression, and prognosis of type 2 diabetes mellitus combined with MASLD. Although animal models can morphologically replicate some of the characteristics of human diabetes combined with MASLD, we also expect the emergence of more ethical genetically humanised animal models. Finally, prospective clinical trial cohorts and more in-depth molecular biology experiments need to be designed and conducted to further validate the mechanism of action of these 2 related genes in the development and progression of type 2 diabetes mellitus combined with MASLD.

The necessity and clinical significance of this study is that, given the current level of medical development for diabetes mellitus combined with MASLD is not yet curable, it is particularly important to consider the active prevention of diabetes mellitus combined with MASLD that has not yet occurred, to improve the quality of survival of more patients, and to reduce the rate of disability and death. Finding the target point of treatment, giving individualised treatment plan and more accurate treatment can reduce the economic pressure of patients, families and even the whole society.

In summary, the candidate genes SPP1 and collagen IV screened based on bioinformatics analysis have the potential to influence the course of type 2 diabetes mellitus combined with MASLD. Through ECM–receptor interaction, Focal adhesion, Human papillomavirus infection, PI3K-Akt signalling pathway, Toll-like receptor signalling pathway signalling pathway, they may play important roles in the course and disease outcome of type 2 diabetes mellitus combined with MASLD, and the results of this study provide meaningful clues and directions for clinical prognosis and treatment.

### Supplementary Information

Supplementary Table 1.

Supplementary Table 2.

## Data Availability

The sequencing data used to support the findings of this study have been deposited in the GEO repository (GSE23343 and GSE49541). The datasets generated and/or analysed during the current study are available in the NCBI repository, and the datasets are available in the GEO repository (GSE23343 and GSE49541). https://www.ncbi.nlm.nih.gov/geo/query/acc.cgi?acc=GSE49541. https://www.ncbi.nlm.nih.gov/geo/query/acc.cgi?acc=GSE23343.
