# Supplementary material for: Diagnostic role of SPP1 and collagen IV in a rat model of type 2 diabetes mellitus with MASLD
Source: Sci Rep. 2024 Jun 17;14:13943. doi: 10.1038/s41598-024-64857-0 (PMC11183142; doi:10.1038/s41598-024-64857-0)
Supplement: Supplementary file 1 — Supplementary Table 1. [file 41598_2024_64857_MOESM1_ESM.doc]

supplementary table1 SPP1 mRNA expression levels

| Factor (n=10) | Statistics description | F | p value |
| --- | --- | --- | --- |
| Group |  | 7.577 | 0.002 |
| Control | 1.335±0.106 |  | a:<0.01, b:<0.05, c:ns |
| DM +MASLD | 1.468±0.045 |  |  |
| HF + HG | 1.368±0.075 |  |  |

# a: DM + MASLD vs Control, b: DM + MASLD vs HF + HG, c: HF + HG vs Control, HF: High Fat diet, HG: High Glucose diet, ns: no significance
