# Supplementary material for: Diagnostic role of SPP1 and collagen IV in a rat model of type 2 diabetes mellitus with MASLD
Source: Sci Rep. 2024 Jun 17;14:13943. doi: 10.1038/s41598-024-64857-0 (PMC11183142; doi:10.1038/s41598-024-64857-0)
Supplement: Supplementary file 2 — Supplementary Table 2. [file 41598_2024_64857_MOESM2_ESM.doc]

supplementary table 2. Collagen Ⅳ mRNA expression levels

| Factor (n=10) | Statistics description | F | p value |
| --- | --- | --- | --- |
| Group |  | 10.049 | <0.001 |
| Control | 1.194±0.041 |  | a: <0.01, b: <0.01, c:ns |
| DM + MASLD | 1.304±0.088 |  |  |
| HF + HG | 1.205±0.04 |  |  |

# a: DM + MASLD vs Control, b: DM + MASLD vs HF + HG, c: HF + HG vs Control, HF: High Fat diet, HG: High Glucose diet, ns: no significance
